# Hematuria as a Diagnostic Clue to Non-cirrhotic Hyperammonemia Due to Corynebacterium urealyticum Urinary Tract Infection: A Case Report

**DOI:** 10.7759/cureus.100082

**Published:** 2025-12-25

**Authors:** Koji Miura

**Affiliations:** 1 General Surgery, Sakaide City Hospital, Sakaide, JPN

**Keywords:** acute hyperammonemia, acute hyperammonemic encephalopathy, corynebacterium spp, corynebacterium urealyticum, gross hematuria, non-cirrhotic hyperammonemia, urease-producing bacteria, uti: urinary tract infection

## Abstract

Hyperammonemia is an important cause of altered mental status in older adults; however, non-cirrhotic hyperammonemia in the absence of underlying liver dysfunction is easily overlooked. Hyperammonemia secondary to urinary tract infection (UTI) caused by urease-producing bacteria is relatively rare, and reliable clinical clues for early diagnosis have not been fully established. We report the case of a 95-year-old woman with Alzheimer’s disease residing in a nursing facility. She had macroscopic hematuria for two days without any change in consciousness. On the day of admission, she was found unresponsive at breakfast and was transported to the emergency department with impaired consciousness. On arrival, she had renal dysfunction and marked hyperammonemia (168 µg/dL) but normal liver function tests. Her urine was alkaline, turbid, and purulent with gross hematuria, and her mental status improved rapidly after bladder catheterization and fluid resuscitation. Urine culture yielded *Corynebacterium urealyticum*, and she was diagnosed with non-cirrhotic hyperammonemia secondary to a UTI caused by this urease-producing organism. Including the present case, a review of 33 English- and Japanese-language reports identified gross hematuria in 15 cases, microscopic hematuria alone in 11, and no hematuria in seven; thus, 78.8% of patients demonstrated some degree of hematuria. These findings suggest that, in older patients with preserved liver function who present with impaired consciousness, the presence of hematuria is an important clue to hyperammonemia secondary to a UTI due to urease-producing bacteria. When hematuria is observed, clinicians should consider this entity in the differential diagnosis and promptly assess urine pH, urinary retention, and indwelling urinary catheters to identify ammonia-producing sources and perform timely drainage to prevent delays in diagnosis and treatment.

## Introduction

Older adults presenting with impaired consciousness are frequently transported to the emergency room (ER). Among the many possible etiologies, hyperammonemia is one that must not be overlooked, yet liver dysfunction is not always apparent from the history or routine blood tests. We encountered a 95-year-old woman who was transferred to the ER because of decreased consciousness following a preceding episode of hematuria and was ultimately diagnosed with non-cirrhotic hyperammonemia secondary to a urinary tract infection caused by the urease-producing organism *Corynebacterium urealyticum*. We report the clinical course of this patient and, based on a review of 32 previously reported cases together with the present case (33 cases in total), we discuss whether hematuria can serve as a diagnostic clue to this condition.

## Case presentation

A 95-year-old woman with Alzheimer’s disease was residing in a nursing facility. Three weeks before presentation, she had fallen and sustained a right femoral neck fracture, for which she underwent surgery at another hospital and was then discharged back to the same facility for rehabilitation and convalescent care. Two days before admission, gross hematuria was noted, but there was no decline in her level of consciousness, and she was observed without further intervention. On the day of admission, when facility staff called to her at the bedside to assist with breakfast, she did not respond and was found to have impaired consciousness. She was transported to our hospital by ambulance.

On arrival, her Glasgow Coma Scale (GCS) score was E1V2M4. Her blood pressure was 156/83 mmHg, heart rate 105 beats per minute, respiratory rate 18 breaths per minute, oxygen saturation 99 percent on room air, and body temperature 37.1 degrees Celsius. Physical examination revealed no abnormal findings other than the decreased level of consciousness.

Blood tests showed leukocytosis with a predominance of neutrophils, renal dysfunction, and marked hyperammonemia (168 micrograms per deciliter), while liver function tests and other serum electrolytes were within normal limits (Table 1).

**Table 1:** Initial laboratory data on arrival at the emergency department Laboratory and arterial blood gas findings on arrival at the emergency department. Arterial blood gas analysis was performed on room air. Values above the institutional reference range are indicated by ↑, and values below the reference range are indicated by ↓. WBC, white blood cell count; RBC, red blood cell count; Hb, hemoglobin; Hct, hematocrit; Plt, platelet count; TP, total protein; Alb, albumin; CRP, C-reactive protein; BUN, blood urea nitrogen; Cre, creatinine; Glu, glucose; T-Bil, total bilirubin; D-Bil, direct bilirubin; AST, aspartate aminotransferase; ALT, alanine aminotransferase; ALP, alkaline phosphatase; γ-GTP, gamma-glutamyl transpeptidase; LDH, lactate dehydrogenase; pCO₂, partial pressure of carbon dioxide; pO₂, partial pressure of oxygen; HCO₃⁻, bicarbonate; tCO₂, total carbon dioxide; BE, base excess.

Parameter	Value	Unit
WBC	12.7↑	×10^3^/μL
RBC	4.36	×10^6^/μL
Hb	12.3	g/dL
Hct	37.9	%
Plt	29	×10^4^/μL
Neutrophil ratio	87.6↑	%
APTT	27.4	s
PT	10.1	s
PT activity	118	%
PT-INR	0.94	–
D-dimer	6.3↑	μg/mL FEU
TP	7.2	g/dL
Alb	3.4↓	g/dL
CRP	6.29↑	mg/dL
BUN	49↑	mg/dL
Cre	1.73↑	mg/dL
NH3	168↑	μg/dL
Glu	133↑	mg/dL
Na	141.7	mmol/L
K	5.6↑	mmol/L
Cl	110.8↑	mmol/L
Ca	9.5	mg/dL
P	4.6↑	mg/dL
T-Bil	0.8	mg/dL
D-Bil	0.2	mg/dL
AST	17	U/L
ALT	9	U/L
ALP	150	U/L
γ-GTP	18	U/L
LDH	222	U/L
pH	7.458↑	–
pCO_2_	24.7↓	mmHg
pO_2_	104	mmHg
HCO_3_^−^	17.3↓	mmol/L
tCO_2_	18↓	mmol/L
BE	-4.8↓	mmol/L
Lactate	0.7	mmol/L

Ultrasonography demonstrated preserved cardiac function without pleural or peritoneal effusion and no abnormalities of the hepatobiliary system. There was no dilatation of either renal pelvis and no evidence of hydronephrosis, but a moderate volume of urine with abundant echogenic debris was retained in the bladder. Urethral catheterization yielded turbid, purulent urine mixed with gross hematuria. Urinalysis revealed a pH of 8.0, protein 3 plus, white blood cells 50 to 99 per high-power field, red blood cells 100 per high-power field, and ammonium magnesium phosphate crystals 1 plus. Head computed tomography showed no abnormal findings.

After bladder catheterization and administration of approximately 1,000 milliliters of intravenous fluids, her level of consciousness improved to GCS E1V3M5. Because of this rapid improvement, cerebrospinal fluid examination and brain magnetic resonance imaging were not performed.

Based on past culture results and the clinical context, the on-call internist initiated treatment with ceftazidime for a presumed urinary tract infection. Given the combination of hematuria, urinary retention, and hyperammonemia, a urinary tract infection due to urease-producing bacteria was suspected. During hospitalization, she was managed with intravenous fluids and an indwelling urethral catheter. By the following morning, her level of consciousness had improved to E3V5M6, and her serum ammonia concentration had normalized to 35 micrograms per deciliter. She was also able to resume normal oral intake.

Several days later, urine culture grew *Corynebacterium urealyticum*, and she was diagnosed with non-cirrhotic hyperammonemia secondary to a urinary tract infection caused by this urease-producing organism. The urethral balloon catheter was once removed, but spontaneous urination did not occur, and she was diagnosed with neurogenic bladder, necessitating reinsertion of the catheter. Although a change of antibiotic to vancomycin was considered based on the culture results, her clinical symptoms and laboratory findings had already improved, so no additional antimicrobial therapy was given, and ceftazidime was discontinued. She was discharged back to the nursing facility on hospital day 12 with the urethral balloon catheter left in place.

## Discussion

Through this case, we considered whether hematuria can serve as a diagnostic clue to non-cirrhotic hyperammonemia secondary to a urinary tract infection caused by urease-producing bacteria and reviewed previously reported cases in the literature. Older adults with impaired consciousness are frequently transported to the ER [1]. Among the many possible causes, hyperammonemia is one that must not be overlooked. However, when liver dysfunction is not evident from the history or laboratory tests, clinicians may be less likely to consider hyperammonemia, and diagnosis can be delayed.

In the present case, we initially suspected septic encephalopathy due to bacteremia originating from a urinary tract infection as the cause of the impaired consciousness. However, the patient’s hemodynamic status was stable, which was inconsistent with this hypothesis. Subsequently, blood tests revealed marked hyperammonemia, and it became clear that hyperammonemia was the main cause of the impaired consciousness. Urinalysis showed alkaline urine, and the combination of hyperammonemia, hematuria, and impaired consciousness in a patient without liver dysfunction suggested the involvement of urease-producing bacteria. Abdominal ultrasonography did not show sufficient urinary retention to indicate severe bladder outlet obstruction, but insertion of a urethral balloon catheter drained approximately 300 milliliters of purulent urine, and her level of consciousness began to improve rapidly over the following hour.

Definitive diagnosis of this condition requires the results of urine culture, which can take time to become available. Therefore, clinical clues that allow early suspicion of infection with urease-producing bacteria are important. In the present case, the clinical course suggested that hematuria could serve as one such useful sign. In previously reported cases, many patients with non-cirrhotic hyperammonemia secondary to urinary tract infection have also been described as having hematuria [2-15], suggesting that hematuria may be a helpful clinical finding for diagnosis in patients with impaired consciousness and preserved liver function. In addition, cases of this condition have been reported in patients with underlying liver cirrhosis [3]. In such patients, it is important not to assume that impaired consciousness is due solely to hepatic encephalopathy but to remain vigilant for concomitant urinary tract infections.

Our review of English- and Japanese-language literature identified a total of 33 cases, including the present case, of non-cirrhotic hyperammonemia secondary to urinary tract infection caused by urease-producing bacteria [2-15]. Of these, gross hematuria was reported in 15 cases, microscopic hematuria alone in 11 cases, and no hematuria in seven cases (Table 2).

**Table 2:** Reported cases of non-cirrhotic hyperammonemia secondary to urinary tract infection with urease-producing bacteria This table summarizes 33 reported cases, including the present case, of non-cirrhotic hyperammonemia secondary to urinary tract infection caused by urease-producing bacteria. “Type of hematuria” is classified as gross, microscopic, or none according to the original reports. “Hospital day of bladder drainage” indicates the hospital day on which bladder drainage (e.g., insertion of an indwelling urethral catheter or intermittent catheterization) was first performed. “Time to improvement in consciousness” represents the number of days from bladder drainage to the first documented improvement in mental status. “Not reported” indicates that the information was not described in the original article. “This case” refers to the current patient in our report. M, male; F, female; BPH, benign prostatic hyperplasia; VUR, vesicoureteral reflux; spp., species; IV, intravenous.

Case no.	Age (years)	Sex	Presenting symptom	Type of hematuria	Associated conditions	Urine pH	Hospital day of bladder drainage	Time to improvement in consciousness (days)	Causative organism	Antibiotic treatment	Reference no.
1	6	M	Impaired consciousness	Gross	Grade IV reflux uropathy	9.0	2	3	Unknown	Ceftazidime	12
2	80	F	Impaired consciousness	None	Chronic urinary retention with hypoactive detrusor	9.0	1	1	Corynebacterium urealyticum	Oral ofloxacin	17
3	44	F	Disorientation	None	Recurrent urinary tract infection	9.0	1	1	*Corynebacterium* species	Not reported	–
4	83	F	Impaired consciousness	None	Neurogenic bladder	Not reported	1	2	Corynebacterium urealyticum	Ceftriaxone	–
5	69	M	Impaired consciousness	Microscopic	Neurogenic bladder	Not reported	2	4	Proteus mirabilis	Ceftriaxone	–
6	80	F	Impaired consciousness	Gross	Not reported	8.0	1	5	Staphylococcus intermedius	Ceftriaxone	9
7	84	F	Impaired consciousness	Microscopic	Hypotonic bladder and several bladder diverticula	8.5	Not reported	Not reported	Corynebacterium pseudodiphtheriticum	Ciprofloxacin	–
8	93	F	Impaired consciousness	None	Neurogenic bladder	8.5	1	1	Corynebacterium urealyticum	Ceftriaxone, vancomycin	19
9	87	F	Impaired consciousness	Gross	Not reported	9.0	1	3	Corynebacterium urealyticum	Levofloxacin, meropenem	6
10	66	F	Impaired consciousness	None	Neurogenic bladder	8.0	Not reported	Not reported	*Escherichia coli*, *Enterococcus faecalis*	Not reported	–
11	98	F	Disorientation and gross hematuria	Gross	Complicated urinary tract infection with urethral stricture	8.0	1	Not reported	Proteus vulgaris	Ceftriaxone	3
12	80	F	Impaired consciousness	Microscopic	Neurogenic bladder	7.5	1	2	Bacteroides ureolyticus	Ampicillin/sulbactam	–
13	71	F	Impaired consciousness	Gross	Parkinson’s disease, drug-induced urinary retention	9.0	1	1	Corynebacterium urealyticum	Ampicillin/sulbactam	14
14	79	F	Impaired consciousness	Gross	Hemorrhagic cystitis, bladder tamponade	8.5	1	2	Corynebacterium pseudodiphtheriticum	Not reported	5
15	88	M	Impaired consciousness	Gross	Neurogenic bladder, BPH	8.5	1	2	*Clostridium* spp., *Bacteroides ureolyticus*	Not reported	11
16	61	F	Confusion	Gross	Neurogenic bladder	8.0	1	Not reported	Proteus mirabilis	Piperacillin/tazobactam	15
17	22	F	Coma	Microscopic	Neurogenic bladder	9.0	1	2	Staphylococcus aureus	Not reported	–
18	66	F	Impaired consciousness	Gross	Not reported	9.0	3	4	Proteus mirabilis	Piperacillin/tazobactam	10
19	86	F	Impaired consciousness	Gross	Neurogenic bladder	Not reported	1	1	Gram-negative rods, Gram-positive cocci	Ciprofloxacin	13
20	60	M	Impaired consciousness	Microscopic	Voiding dysfunction	Not reported	1	2	Staphylococcus saprophyticus	Not reported	–
21	83	F	Impaired consciousness	Microscopic	Neurogenic bladder	7.5	Not reported	5	Staphylococcus aureus	Not reported	–
22	88	F	Impaired consciousness	Microscopic	Neurogenic bladder	8.5	Not reported	5	Actinobaculum urinale	Not reported	–
23	94	F	Impaired consciousness	Gross	Neurogenic bladder	Not reported	1	2	Urease-producing Corynebacterium species, coagulase-negative *Staphylococcus aureus*, *Escherichia coli*	Cefcapene pivoxil	7
24	10	F	Impaired consciousness	Gross	Bilateral VUR	8.0	1	1	Morganella morganii	Details not described	4
25	88	F	Impaired consciousness	Gross	Hemorrhagic cystitis	9.0	1	2	Coagulase-negative staphylococci	Ceftriaxone, meropenem	8
26	87	F	Impaired consciousness	Microscopic	Dementia with Lewy bodies	9.0	4	5	Corynebacterium urealyticum	Ampicillin/sulbactam	–
27	69	M	Impaired consciousness	None	Acute urinary retention	Not reported	1	Not reported	Proteus mirabilis	Not reported	–
28	80	F	Impaired consciousness	Microscopic	Voiding dysfunction	8.0	4	5	Corynebacterium riegelii	Cefmetazole, vancomycin	18
29	72	F	Impaired consciousness, vomiting	Gross	Urinary tract infection with urinary retention	Not reported	3	6	*Escherichia coli*, *Klebsiella pneumoniae*	Not reported	2
30	69	M	Impaired consciousness	None	Bladder outlet obstruction	Not reported	1	2	Proteus mirabilis	Oral rifaximin plus intravenous meropenem and vancomycin	–
31	54	F	Impaired consciousness	Microscopic	Severe vulvar edema	8.0	17	22	*Proteus* spp.	Levofloxacin	–
32	95	F	Impaired consciousness	Gross	Neurogenic bladder	8.5	1	2	Corynebacterium urealyticum	Ceftazidime	This case
33	85	F	Impaired consciousness	Microscopic	Neurogenic bladder	8.5	1	1	Corynebacterium riegelii	Ampicillin/sulbactam	–

Thus, some degree of hematuria was present in 78.8% of patients, whereas gross hematuria was observed in only about 45% of cases, and approximately one-fifth of patients showed no evidence of hematuria at all. On the basis of this analysis, in patients with impaired consciousness accompanied by hematuria, non-cirrhotic hyperammonemia due to urinary tract infection caused by urease-producing bacteria should be considered in the differential diagnosis. Although hematuria can be regarded as a useful clinical sign that raises suspicion for this condition, clinicians should also bear in mind that some cases may lack hematuria. In addition, urinary tract infections caused by urease-producing bacteria are known to be associated with increased urinary ammonia and alkaline urine [16]. Therefore, when hematuria is observed in patients with impaired consciousness, it is advisable to check the urine pH.

In the present case, hematuria was evident from the history, but there was no obvious urinary retention. Abdominal ultrasonography showed a moderate amount of urine, approximately 300 milliliters, with abundant debris in the bladder. The patient had been recuperating at home after surgery for a femoral trochanteric fracture and had decreased activities of daily living, which likely reduced the frequency of voiding. We speculate that residual urine became infected with *Corynebacterium urealyticum*, resulting in increased urinary ammonia. Venous blood containing ammonia absorbed through the bladder wall can bypass the portal circulation and the liver and flow directly into the systemic circulation [17]. Consequently, blood ammonia levels may rise, and encephalopathy can develop.

Previous reports have described cases in which this condition was not initially suspected, but placement of a urethral catheter for management of urinary retention or impaired consciousness on the ward incidentally resulted in effective drainage and clinical improvement. With regard to treatment, relief of urinary tract obstruction is the most important initial step and can be expected to reduce blood ammonia levels promptly. In our patient as well, drainage and fluid resuscitation alone led to rapid improvement in the level of consciousness. Because urine culture results require time to become available, definitive diagnosis of this condition often takes several days. In some reported cases, diagnosis required up to four days and initiation of treatment was delayed, whereas in others, early drainage led to rapid improvement, underscoring its diagnostic and therapeutic importance [18,19]. From the standpoint of preventing recurrence, appropriate treatment of cystitis and correction of underlying voiding dysfunction are also essential.

## Conclusions

Based on this case and a review of 33 cases reported in the literature, including the present case, hematuria and alkaline urine in older patients with impaired consciousness and no obvious liver dysfunction may serve as important clues to non-cirrhotic hyperammonemia secondary to urinary tract infection caused by urease-producing bacteria, including *Corynebacterium urealyticum*. In such patients, clinicians should not assume hepatic encephalopathy alone but should also consider a possible association between urinary tract infection and hyperammonemia from an early stage.

In particular, confirming urine pH and promptly identifying conditions that can serve as sources of ammonia production, such as urinary retention and indwelling urinary catheters, followed by timely drainage and other appropriate interventions, are essential to prevent delays in diagnosis and treatment. These measures are also likely to contribute to improved outcomes in older patients presenting with similar clinical conditions.
